# A rapid and simple protocol for the isolation of bacteriophages from coastal organisms

**DOI:** 10.1016/j.mex.2019.11.003

**Published:** 2019-11-07

**Authors:** Alex Echeverría-Vega, Pablo Morales-Vicencio, Camila Saez-Saavedra, Felipe Gordillo-Fuenzalida, Rubén Araya

**Affiliations:** a Vicerrectoría de Investigación y Postgrado, Universidad Católica del Maule, Avda. San Miguel 3605, Talca, Chile; b Laboratorio de Microbiología Costera, Facultad de Ciencias del Mar y Recursos Biológicos, Universidad de Antofagasta, Angamos 601, Antofagasta, Chile; c Centro de Biotecnología de los Recursos Naturales, Facultad de Agronomía y Ciencias Forestales, Universidad Católica del Maule, Avda. San Miguel 3605, Talca, Chile; d Instituto de Ciencias Naturales Alexander von Humboldt, Facultad de Ciencias del Mar y Recursos Biológicos, Universidad de Antofagasta, Angamos 601, Antofagasta, Chile

**Keywords:** Isolation of bacteriophages from coastal zone, Vibrio bacteriophage, Phage prospection, Phage isolation

## Abstract

This work details a protocol for recovering bacteriophages from intertidal sessile mussels and testing their lytic activity against pathogenic bacteria. Although bacteriophages were highly abundant in coastal filter-feeding organisms, they were not detectable in the surrounding water column. This difference reflects the high filtering rate of the mussels, which capture and concentrate high amounts of bacteria, generating an ideal environment for bacteriophages. We validated the protocol providing a mean concentration of 4E + 04 PFU mL^-1^ lytic bacteriophages for the fish-pathogen bacterium *Vibrio ordalii*. We suggest that this method has particular utility for the recovery of bacteriophages for use as natural antimicrobial agents in aquaculture.

## Graphical abstract

**Figure:**
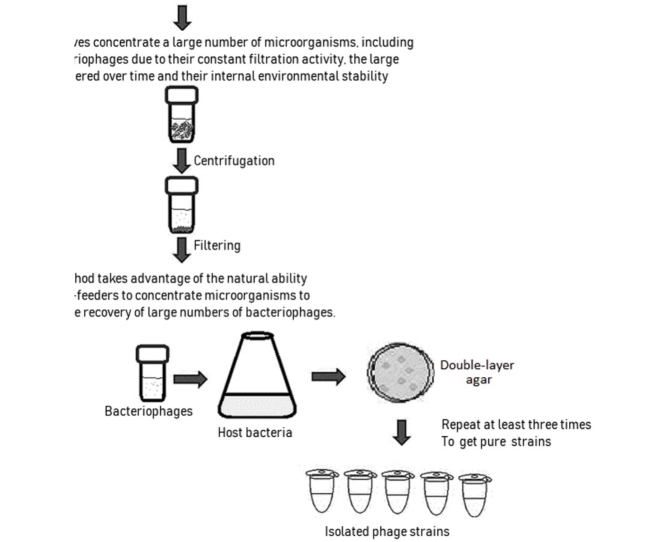

**Specification Table**

**Table:** 

Subject Area:	Immunology and Microbiology
More specific subject area:	Bacteriophages
Protocol name:	Isolation of bacteriophages from coastal zone
Reagents/tools:	DAPI flourescent dye (4 ',6-diamine-2-fenilindole-dilactate, Merck) Paraformaldehyde (Sigma-Aldrich) Tryptone-soy agar (TSA) and tryptone-soy broth (TSB) (Thermo, Oxoid) Polycarbonate membrane filters, 0.22 μm pore size (Merck, Millipore) Vecta Shield mounting medium (Vector Labs) Bacto-Agar (Difco) fluorescence microscope using blue filter set (λ excitation ∼ 359 nm, λ emission ∼ 461 nm), with100x lens with an standardized 10 × 10 μm grid in the eyepiece Zeiss (Axiolab) Water bath (Memmert WNB 7) Microplate Spectrophotometer UV-Vis Epoch (BioTek Instruments, Inc.) Incubator (Memmert IN 75) Centrifuge (Hermle Z 320) Microcentrifuge (Hettich Zentrifugen Mikro 200R) Laminar flow hood (ESCO Biobase BBS-V800)
Experimental design:	Benthic filter-feeding mollusks were collected from the coastal zone of Antofagasta, Chile. Soft tissues were extracted and homogenized. The product was sequentially centrifuged and filtered to eliminate large particles. Chloroform was added to remove bacterial remnants and a virion suspension was carefully recovered. Lysis tests and bacteriophage counts were further performed by plate assays.
Trial registration:	Not applicable
Ethics:	Not applicable

**Value of the Protocol**

**Table:** 

• A method with high efficiency for the recovery of bacteriophages with the capacity to lyse bacterial cells from coastal zones. • Large number of bacteriophages recovered from limited sample mass. • Inexpensive, fast and easy methodology

## Description of protocol

### Background information

**Fig. 1:**
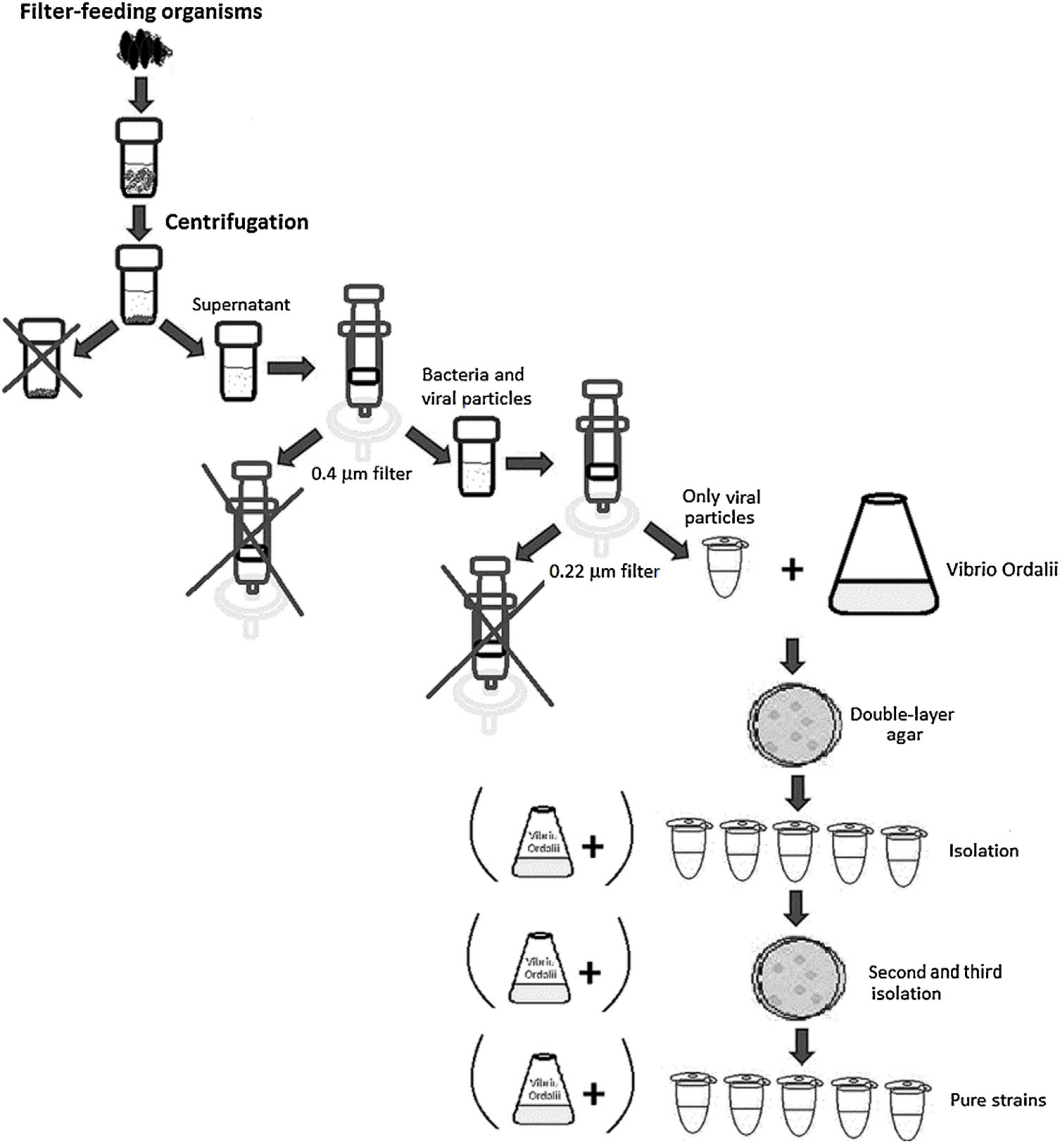
Schematic of phage isolation from filter-feeding organisms.

Although large numbers of bacteriophages are reported from seawater, the successful recovery of bacteriophages capable of killing specific pathogenic bacteria is dependent on host density and surrounding environmental conditions. Most existing protocols to recover lytic bacteriophages from coastal zones require large volumes of water inoculated with host bacteria to enrich the target bacteriophages followed by the concentration of viral particles through tangential flow filtration or by ultracentrifugation [1,2]. These methods lack efficiency when bacteriophage numbers are low. Conversely, filter feeding bivalves such as mussels concentrate a large number of microorganisms, including bacteria and bacteriophages due to their constant filtration activity, the large volume of water filtered over time and their internal environmental stability. Our method takes advantage of the natural ability of filter-feeders to concentrate microorganisms to allow the recovery of large numbers of bacteriophages using a quick and easy protocol (See Fig. 1). We have validated our method by searching for specific bacteriophages capable of lysing the bacterium *Vibrio ordalii* from the Chilean coastal zone. We selected an abundant filter-feeding intertidal mussel (*Perumytilus purpuratus*) and recovered a high number of bacteriophages. *Vibrio ordalii* was grown at 20 °C in tryptone-soy agar (TSA) or tryptone-soy broth (TSB) (Thermo, Oxoid) in which 50 % of the water was replaced with filter-sterilized seawater.

### Bacterial counts

To count the bacterial cells in the suspension, the sample was treated with DAPI fluorescent dye (4′,6-diamine-2- phenylindole-dilactate, Merck) [3]. After DAPI staining, three 10 mL serial dilutions (1/10, 1/100 and 1/1000) were performed and 4 % paraformaldehyde was added to each. The solutions were filtered using 0.22 μm pore size polycarbonate membranes (Merck, Millipore), and the filters were allowed to dry for 15 min over absorbent paper. 15 μL of a 2 mg/L DAPI solution, was placed on a slide, covered with the filters and incubated in a dark damp box for 15 min at room temperature. The filters were removed and washed in 96 % ethanol for 10 s and further dried on absorbent paper for one hour at 37 °C in the dark. Dry filters were mounted on a slide with 10 μL of Vecta Shield mounting medium (Vector Labs) and covered with a clean cover glass. Stained cells were observed in a fluorescence microscope with a filter set of ∼359 nm λ excitation and ∼461 nm λ emission, using a 100x lens with a standardized 10 × 10 μm quadrant in the eyepiece as counting grid. A minimum of 500 cells was counted for each sample and the dilution was chosen that allow the required fields to be stated. The total number of cells per sample was estimated by establishing the ratio between counting area and the total effective filtration area of the polycarbonate filter. This value was multiplied by the number of counted cells and the dilution factor and divided by the total filtered volume as showed in the equation:

(A*X[cells]*f) (a*1[mL])^-1^

where, **A** is the filtration area; **X** is the number of counted cells; **f** is the dilution factor, and **a** is the area of the field observed under the microscope.

### Isolation of bacteriophages

Benthic filter-feeder mollusks (mytilid mussels) were collected from the coastal zone of Antofagasta, Chile. These were placed in sterile containers and processed on the same day of collection. First, 100 g of soft tissue were extracted from the shells and homogenized, adding 100 mL of seawater into a sterile blender for 1 min. The product was centrifuged for 10 min at 4000 g and ∼ 50 mL of supernatant was carefully collected, avoiding the movement or inclusion of solid debris. At this stage, a viscous supernatant was obtained due to the presence of elevated amounts of suspended solids, in addition to microorganisms and viral particles. Bacterial counts were conducted during this stage. Two mL of supernatant was sequentially filtered through nitrocellulose membranes of 5 μm, 0.45 μm, and 0.22 μm pore size (Millipore) to eliminate major particles. 100 μL of chloroform was added to 1 mL of the filtrate to remove the remaining bacterial remnants. After 30 s of vortex shaking, the tubes were centrifuged for 5 min at 10,000 *g*. Finally, 500 μL of the aqueous phase corresponding to the virion suspension was recovered, carefully avoiding the uptake of chloroform.

### Bacteriophage counting

Specific lytic phages were isolated by the double layer agar culture technique [1,4]. For this, 10 mM of MgCl_2_ was added to the culture medium (TSB), to provide the divalent cations required for phage multiplication [1]. Two-layer gel-plates were prepared using Bacto-Agar (Difco) as a gelling agent at two different concentrations: 1.5 % for the lower layer and 0.7 % for the upper layer. The lower layer media was autoclaved and poured into Petri dishes. The upper layer media was autoclaved and maintained in glass tubes at 45 °C prior to use (for no more than 1 h). For the cultivation of phages, a bacterial inoculum in the exponential phase was prepared. Subsequently, three serial dilutions of the enriched (putative) virion samples (1/10, 1/100 and 1/1000) were prepared and 1 mL of each of them was mixed with 0.1 mL of the bacterium culture in the early exponential phase and 3 mL of the 0.7 % agar. The mixtures were homogenized and poured onto previously prepared plates with the solid (low layer) agar. Finally, the plates were incubated overnight at 20 °C, after this time, the lysis plates counts were performed, establishing the number of plates forming units (PFU) for each dilution.

### Selection of lytic phages

At least 30 clearly defined lysis plates were selected by gently picking them with sterile wooden sticks (toothpicks), and used to inoculate 1 mL of early exponential bacterial culture containing 10 mM MgCl_2_. They were incubated overnight at 20 °C and the isolation process was repeated at least three times to ensure the presence of a single lytic phage per tube. The phages obtained from the process were named according to the nomenclature norms proposed by Kropinski et al. [5], following the detail published in the REBASE database (http://rebase.neb.com/rebase/rebase.html). Presence of genes associated with a temperate life cycle (i.e. integration/excision genes) must be evaluated by molecular techniques (PCR-Sequencing) to finally ensure that our isolated is an obligately lytic bacteriophage [6].

### Enrichment and phages selection

As an optional step to increase the number of specific phages for a bacterium, 200 μL of the suspension of (putative) virions could be added to 20 mL of early exponential bacterial culture, supplemented with 10 mM of MgCl2. Then, keep it at 20 °C until observing a medium clarification, for a maximum period of 48 h. To recover the phages, follow the procedure described previously.

### Validation

We undertook a search for lytic bacteriophages for the bacterial strain *Vibrio ordalii* ATCC-33509 in the intertidal zone of San Jorge bay (Antofagasta, Chile, 23°38′00″ S 70°24′00″ W). We sampled at five points along a 50 m transect, with each point separated by 10 m. At each point, 5 L of sea water and 20 bivalve mussel (*Perumytilus purpuratus*) were collected and subsequently processed to assess bacterial counts and lytic phage recovery for *Vibrio ordalii*, following the protocol detailed above. Water samples were subject to a prior enrichment stage to increase the number of lytic specific phages for *Vibrio ordalii*. Mean (± SD) cell counts were 1E + 09 (± 2E + 08) cell/mL in *Perumytilus* and 7E + 5 (±3E+05) cell/mL in the surrounding sea water.

**Fig. 2:**
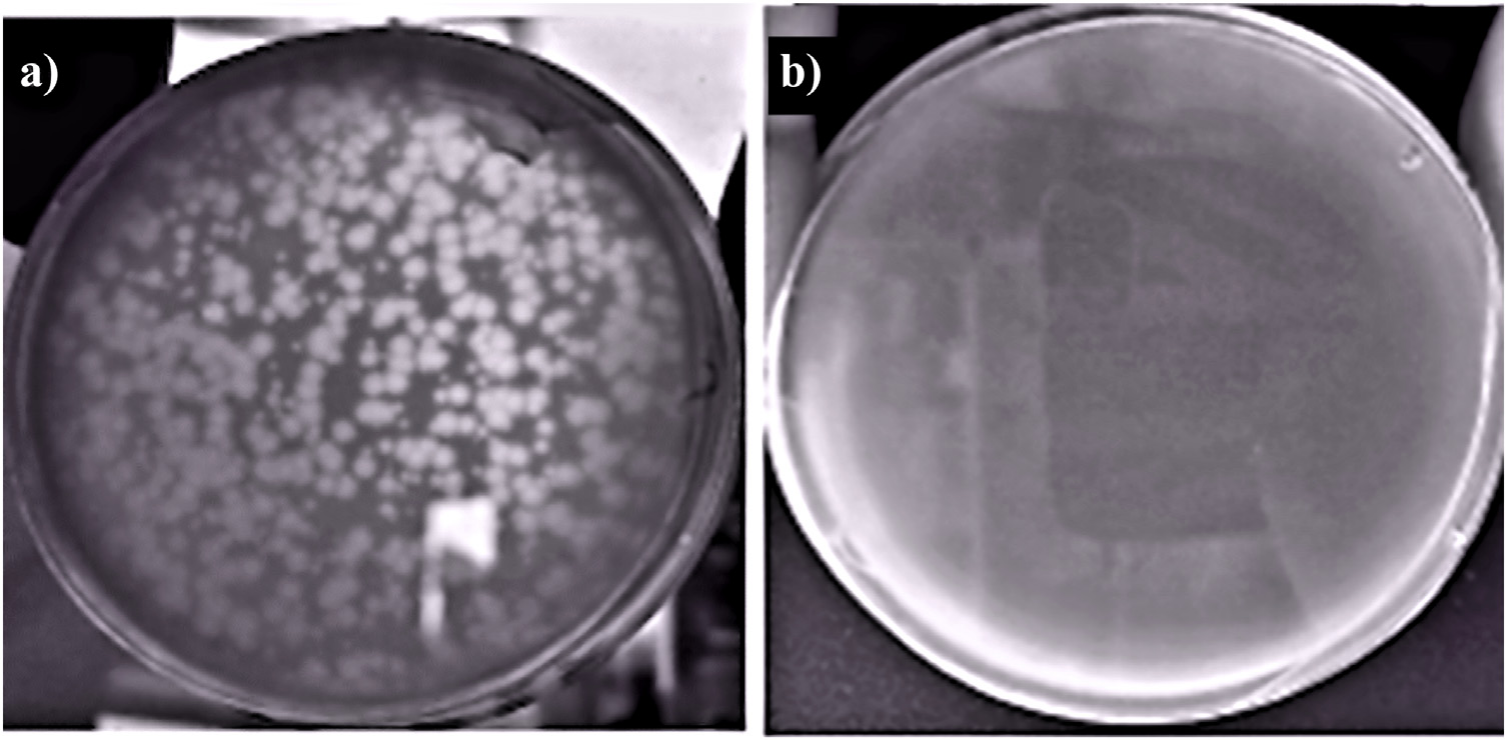
Double layer agar plate with phage cultures from a) *Perumytilus purpuratus* extract (dilution 1:100); b) Sea water. Notice the presence of lysis plaques only in a).

Lytic activity against *Vibrio ordalii* was observed for all the extracts from *Perumytilus*, with a mean (±SD) total count of lysis plaques, of 4E + 4 (±8E+03) PFU/mL (Fig. 2). No lysis plaques were observed from seawater samples (Fig. 2b). A total of 30 well-defined lysis plates were selected and separated from the rest (Fig. 2a). Seven of the selected plaques (putative phages) still showed lytic activity after the third re-inoculation. We selected one of these re-inoculated plaques to be sequenced and analyzed, resulting in the identification of a lytic bacteriophage belonging to the Siphoviridae family [7]. Phages with the capacity to lyse other native bacterial strains previously isolated from mussels were also found and are currently being evaluated.
